# On top of that all, now Covid-19, too. A scoping review of specificities and correlates of fear of cancer recurrence in breast cancer patients during COVID-19^☆^

**DOI:** 10.1016/j.breast.2022.02.007

**Published:** 2022-02-10

**Authors:** Éva Kállay, Flavia Medrea, Csaba László Dégi

**Affiliations:** a Psychology and Educational Sciences, Babes Bolyai University, Cluj Napoca, Romania; b Sociology and Social Work, Babes Bolyai University, Cluj Napoca, Romania

**Keywords:** Fear of cancer recurrence, Fear of cancer progression, Breast cancer, Risk factors, Covid-19 pandemic

## Abstract

Fear of cancer recurrence (FCR) is a normal response in cancer survivors and one of the most prevalent reactions reported by up to 87% of them. However, elevated levels of FCR impair well-being, quality of life and professional functioning, and lead to anxiety, depression or PTSD. COVID-19 pandemic can exacerbate FCR symptoms, given the restricting access to follow-up investigations and treatment, the isolation restrictions imposed and the possibility of the medical system becoming overworked. This scoping review's objective was to synthesize the literature investigating the factors associated with higher levels of FCR in cancer survivors during the COVID-19 pandemic. The focus was on FCR in breast cancer patients, including most of the studies (5 out of 9) on this topic. However, given the novelty of the subject, the increased interest in it, and the fact that there are few studies in this field, the review included 4 other studies with mixed samples of patients with breast cancer and other oncological pathologies. Following rigorous methodological criteria, 9 studies with quantitative or mixed methodology were included (N = 4831 patients). The results indicate that high levels of FCR are associated with distress and concerns regarding the pandemic impact, with most common concerns of patients being changes in treatment plan (delays and interruptions), dysfunctional communication with medical staff or difficult access to food or medicine. The most common correlates of FCR during the pandemic are marital status, childlessness, low financial status, level of education, type of cancer diagnosis, generalized anxiety and depression.

## Highlights

• The context of the COVID-19 pandemic can exacerbate FCR symptoms.
• High levels of FCR are associated with distress and concerns regarding the impact of the pandemic.
• The most common concerns indicated by patients are: changes in treatment plan (delays and interruptions), dysfunctional communication with medical staff, and difficult access to food or medicine.
• Among correlates of FCR during the pandemic are marital status, low financial status, generalized anxiety, and depression.

## 1 Introduction

Cancer-related diseases represent one of the leading causes of death before the age of 70, being also an important factor that lowers life expectancy in almost every country in the world [1,2].

The diagnosis with cancer itself is usually considered an extremely stressful, potentially traumatic encounter [3,4]. First and foremost, the multiple physical implications of the disease (the discomfort produced by the tumor treatment: surgery, radio and/or chemotherapy, acute and chronic pain, fatigue, lymphedema, etc.) [5] significantly affect the patients' well-being and quality of life [6,7]. The impact of the diagnosis and treatment simultaneously affect the psychological, social, spiritual, professional functioning of the patients, and their proximal and distal environments as well (families, relatives, friends, co-workers, etc.) [8]. Over one-third of the patients in acute care present significant psychological disorders (e.g., anxiety, depression, adjustment disorders, posttraumatic stress-disorder) [9,10], and the associated social and economic cost are by no means negligible [11]. In a considerable number of cases, the entire cancer-experience may be considered as a situation of multiple traumatic encounters in which the diagnosis would represent one of the traumatic factors.

Moreover, the entire cancer experience is infused with an emotion which constantly pendulates between background and foreground psychological states, namely, fear of cancer recurrence (FCR). FCR is usually defined as “*fear, worry, or concern about cancer returning or progressing*” [12] [p. 3].

## 2 Fear of cancer recurrence

FCR is a totally normal reaction to the threats involved in the complexity of aversive confrontations patients have to undergo since diagnosis [13]. Simard et al.'s [14] systematic review indicates that regardless of the cancer type and assessment method, between 39% and 97% of cancer survivors reported varying degrees of FCR: 22%–87% of the assessed patients indicated that have experienced moderate to high levels of FCR, and 0%–15% reported experiencing high degrees of FCR.

FCR may appear at any stage of the illness trajectory, and may persist many years even after treatment or remission of illness, being relatively stable over time [[15], [16], [17]]. One of the central aspects of FCR is the uncertainty of the prognostic, which, in many cases is significantly associated with maladaptive behavioral, emotional, and cognitive responses. These dysfunctional reactions are further associated with heightened levels of fear, worry, exaggerated vigilance regarding possible symptom recurrence or on the contrary, behavioral avoidance, etc. [18].

Even if FCR shares some common characteristics with different anxiety disorders, it should be qualitatively differentiated from them, since in the case of oncological illnesses the threats are real, and the patients' fear cannot be considered as irrational, as is the situation in the case of neurotic anxiety disorders for example [13].

So, to some degree FCR is a normal and adequate reaction to the physical and psychological challenges and uncertainties oncological patients must face after being diagnosed [19]. However, persistent, elevated levels of FCR may become a serious impediment in the face of adaptation, leading to dysfunctional reactions [13].

Literature indicates that those cancer patients who experience high levels of FCR may experience additional maladaptive changes, as: impaired well-being, quality of life, and professional functioning; difficulties in making plans for the future; intrusive thinking; extremely frequent checking of different signs of cancer (hypochondria); excessive use of health care services; development of anxiety disorders; posttraumatic stress disorder; depression, etc. [[20], [21], [22], [23], [24]]. Furthermore, high levels of FCR may become a serious, chronic problem not only for the affected patients themselves [25], but may also negatively impact the life of their caregivers [26].

Since, worldwide, the majority of the cancer survivors consider that FCR is one of their most serious unmet need, it became imperative to identify those patients who are at risk to develop and maintain maladaptive levels of FCR, and to develop prevention and intervention strategies to reduce dysfunctional FCR [27].

## 3 Risk factors of FCR

A theoretic [19] and several systematic [14,27] reviews conducted on this topic have identified essential risk factors that predispose some patients to experiencing higher levels of FCR, the most prevalent ones falling into the following categories: socio-demographic, physical and psychological.

Of the **socio-demographic risk factors,** younger age was found to be systematically associated with higher levels of FCR [14,19,27]. Studies investigating the importance of gender, level of education, marital status, income, ethnicity, and employment yielded mixed results regarding the development and maintenance of FCR [14,27]. However, a number of studies found that female patients seem to be more prone to experience FCR than male cancer survivors [[27], [28], [29]].

Of the most frequently investigated **physical factors** related to FCR (e.g., time since diagnosis, site of cancer, severity, stage of illness, treatment type, type of surgery), strong evidence was found between high levels of FCR and presence/severity of physical symptoms, side-effects of treatment, pain, and receipt of chemotherapy [14,19,27]. The investigation of the rest of the physical risk-factors produced mixed results.

Regarding **psychological risk-factors**, prior history of mental health problems, as previous exposure to traumatic situations (subclinical, clinical PTSD), different anxiety disorders, mood disorders emerged as stable risk factors for FCR [19]. Neuroticism was found to be strongly related to higher levels of FCR, while self-esteem, healthy coping, sense of coherence tended to lower FCR [14,19,27].

Moreover, exposure to media information, pending medical appointments, hearing of other people being also diagnosed with cancer, experiencing new or changing side-effects of the treatment or intensification of pain influences and increases reports of FCR [24,27,30,31].

Of utmost importance is the fact that the certainty of access to periodic follow-up examinations were reported to reduce the experience of FCR [32], while disruption of access to healthcare services was found to increase worry and concerns about the progression and recurrence of the disease [33].

## 4 The Covid-19 pandemic

The 2019 coronavirus outbreak has massively changed human life all around the world. Due to its rapid spread, high levels of contagion and serious, multiple impact on human health, by the beginning of March 2020, the WHO had to declare COVID-19 a worldwide pandemic [2,34]. Literature investigating human reactions during pandemics in general indicated that such multiple threats are usually accompanied by high levels of stress, anxiety, uncertainty, loneliness, confusion regarding one's and close ones' health, financial concerns, the possibility to lose loved ones, etc. could be considered as extremely stressful situations [[35], [36], [37]].

In the general population, the already high levels of stress concerning the infection with COVID were aggravated by the conditions created by the quarantine. The isolation from family, friends, and co-workers, the permanent threat that health-care systems might be overwhelmed by the increasing number of infected patients, disruptions of usual life-routines, the negative psychological effect of the curfew, changing work habits (e.g., telework), etc. further aggravated the initial stressors, significantly affecting the populations' emotional and mental well-being [38,39].

## 5 The Covid-19 pandemic and cancer survivors

The objective risk of contamination with the COVID-19 is significantly higher for cancer patients whose immunity is usually affected by either the disease itself or the afferent treatment [40,41].

If we take into consideration the specific situation of cancer survivors in normal life-conditions, it becomes obvious that the impact of the COVID-19 pandemic has further aggravated their already fragile situation. Knowing on the one hand that cancer has been a very serious independent risk factor for in-hospital mortality among patients diagnosed with cancer [42], and on the other hand that access to follow-ups and treatment was seriously restricted, it could be expected that FCR might have been exacerbated by the accumulating pandemic-induced emotional upheaval.

## 6 Objective

Thus, the major aim of the present paper was to review the currently available literature which examines factors that are associated with higher levels of FCR in breast cancer and other oncological pathologies survivors during the Covid-19 pandemic.

Based on the existing literature and taking into consideration the extremely large variety of types of cancer [43], each purporting different mortality and survival rates, physical, psychological, social, professional, etc. implications, we may consider that the stable risk factors in normal life conditions for increased FCR for most forms of cancer are: younger age, presence and severity of symptoms, side-effect of treatment, pain, treatment with chemotherapy, previous traumatic encounters, psychological disorders as: anxiety, mood disorders, and high levels of neuroticism. Moreover, disruptions in the access to health-care services, pending medical appointments, new or changing side-effects of treatment, intensification of pain may further exacerbate FCR [14,19,27].

Literature has also identified that higher levels of self-esteem, frequent use of different adaptive coping mechanisms, sense of coherence and certainty of access to periodic follow-ups have a benefic influence on the entire cancer experience, simultaneously reducing FCR [14,19,27].

## 7 Methods

In order to rigorously answer the present study's major objective, we opted to conduct a scoping review of the topic of FCR in breast cancer and other oncological pathologies survivors during the Covid-19 pandemic. The methodology of a scoping review offers the possibility to systematically map the literature investigating a specific issue [44], and to summarize evidence, and identify possible knowledge gaps [45]. According to Peters et al. [46]; this type of investigation is very useful, when literature is characterized by high complexity and heterogeneity, which is the case of the topic selected by us to investigate. Moreover, the results of a scoping review may be highly informative for decision-makers by assisting the development of intervention agendas [46].

## 8 Search strategy

The present review was conducted according to the PRISMA-ScR (Preferred Reporting Items for Systematic Reviews and Meta-Analyses extension for Scoping Reviews) guidelines appropriate for scoping reviews [46,47]. An electronic literature search was conducted in order to identify all studies published between January 2020 and up to October 1, 2021, across sources, including Web of Science, PubMed, Cochrane library, Medline, PsycINFO, Scopus, Science Direct. The time range was limited as related to the period of time of the COVID-19 pandemic, its beginning, and the moment when a sufficient number of studies have been published, so we could conduct a scoping review (October 1, 2021). Terms associated with fear of cancer recurrence (FCR), worry of cancer recurrence, concern of cancer recurrence, fear of cancer progression, worry of cancer progression, and concern of cancer progression were selected. References in the identified papers have also been checked and reviewed. The searches were conducted between 1st of July and October 1, 2021.

## 9 Eligibility criteria

Thus, in order to attain our research objective, we included in our review full-length articles published in international peer-reviewed journals, based on the following inclusion criteria:

● To be written in English
● To be published on the topic of fear of cancer recurrence (FCR) and fear of cancer progression (FCP) during the Covid-19 pandemic or sub-components of these concepts (fear, worry, concern of cancer recurrence or progress) (*Covid-19, *Corona-virus pandemic, *Sars-Cov-2)
● To include adult population of cancer survivors diagnosed with any type of cancer
● To use quantitative or mixed methods of investigation
● Review articles, book chapters, editorials, poster abstracts, case reports, commentaries, and dissertations were excluded

**Fig. 1:**
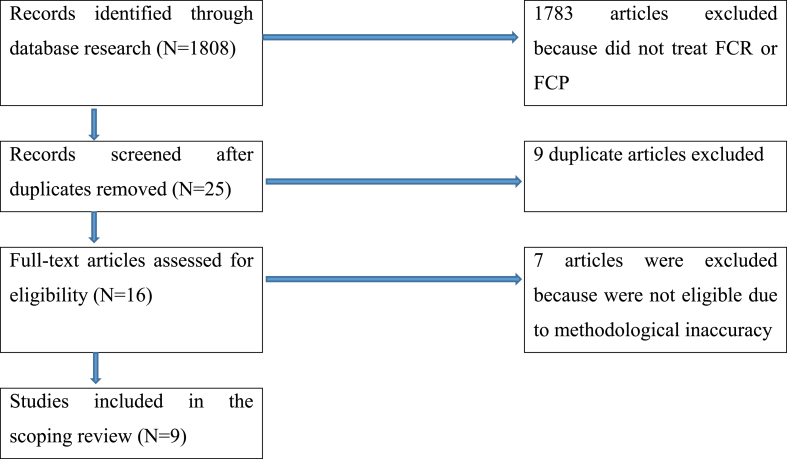
Search process.

The selection process of the relevant literature of this scoping review is graphically presented in Fig. 1. The initial search with the above-presented key-words yielded 1808 studies. After removing studies that did not directly research the key-words of interest, we remained with 25 studies. After excluding 9 duplicate-studies we subjected to eligibility assessment 16 investigations, and after excluding 7 articles for not fulfilling the standards of methodological accuracy, we included in our scoping review 9 articles.

Each of the 9 selected articles subjected to scoping review was assessed regarding its quality, by two independent reviewers regarding: country where the study was conducted, sample size, stage and type of cancer, type of study (qualitative, mixed – qualitative studies were excluded), time since diagnosis, measures of fear of cancer recurrence/progression, results, factors/variables associated with FCR/FCP, possible biases and methodological limitations. Results are presented in Table 1.

**Table 1:** Description of eligible studies.

No.	Study, country	Study design	Sample size, Cancer type	Measures of FCR	Other variables usually associated with greater FCR, also measured in the study	Results	Limitations of the study
1	[48] South Korea April–June 2020	Descriptive correlational Cross-sectional Quantitative Qualitative	**N = 154** Breast cancer survivors	**Instruments** Fear of Cancer Recurrence Inventory (K-FCRI) subscales: triggers severity psychological distress coping strategies functional impairments insight reassurance **validated instrument**	Age Marital status Nr of children Nr of children living together Ages of children living together Level of education Monthly income Economic burden Area of residence Timing of diagnosis Treatment type Time required to visit medical institutions Experience of quarantine due to Covid-19 suspicion Whether diagnosed with Covid-19	FCR high (84.31 ± 24.23) Triggers (21.30 ± 6.50) Coping strategies (20.58 ± 6.09) Severity Demographic variables FCR significantly higher among unmarried (F = 3.649, P-.028) Without children (t = −2.043, P = .043) Radiotherapy (t = 2.790, P = .006) Monthly income (F = 4.259, P = .016)	Convenience sample, Online assessment Selection bias Restricted access to rural areas South Korea – high quality of health insurance and medical infrastructure Did not exclude patients with psychiatric diagnoses No investigation on categories of FCR Not controlling of variables that could have affected psychological status
2	[33] Turkey 10–20 May 2020	Descriptive cross-sectional online survey	**N = 82** female, non-metastatic breast cancer survivors	**Instruments** Fear of Cancer Recurrence Inventory-Short Form (FCRI-SF)– Turkish validated form 12-item Spiritual well-being (SWB) scale (Peterman et al., 2002) – adapted to Turkish 6-item brief resilience scale (BRS) (Smith et al., 2008) – Turkish validated form	Demographic variables: - marital status - age Smoker-non-smoker - income	84.1% of the participants experienced high levels of FCR Variance analyses did not indicate any statistically significant differences in FCR depending on the assessed demographic variables Correlations between FCRI-SF and BRS and SWB Hierarchical linear regression analysis SWB affects FCRI-SF Scores Mediation analysis indicated that SWB plays a partial mediating role in the relationship between BRS and FCR	A very important limitation of this study was that it excluded cancer patients older than 50 years of age
3	[49] Canada 28 April and May 29, 2020	Cross-sectional study Online assessment (a secondary analysis of a larger ongoing longitudinal study)	**N = 36** non-metastatic breast cancer survivors	**Instruments** 7-item Insomnia Severity Index (ISI) 14-item HADS Hospital Anxiety and Depression Scale The 9-item Severity Subscale of the Fear of Cancer Recurrence Inventory (FCRI) Scores higher than 13 indicate clinical levels of FCR **Inclusion criteria** (a) non-metastatic breast cancer diagnosis, (b) scheduled to receive chemotherapy in the upcoming days/weeks, (c) between 18 and 80 years of age, and (d) able to read and understand French	Age Metastatic-non-metastatic cancer Scheduled to receive chemotherapy 14-item COVID-19 stressors questionnaire (e.g., difficulty obtaining needed help or social support) plus 1 - item possibility to contract Covid-19 2 – items regarding the possibility that cancer may progress or be less likely to be cured due to changes in cancer treatment	of the assessed participants 44.4% reported clinically significant levels of anxiety, 41.7% insomnia 52.8% FCR and 16.7% reported clinically significant levels of depression higher levels of concerns regarding the implication of Covid-10 pandemic was significantly associated with FCR, anxiety, depression, and insomnia.	Low number of participants Study conducted at the very beginning of the pandemic
4	[50] USA 27 June – August 13, 2020	Cross-sectional study Online assessment	**N= 50** female cancer survivors with dual carcinoma *in situ*, lobular carcinoma *in situ*, or invasive breast cancer whose cancer surgery was postponed due to the pandemic Non-metastatic breast cancer	**Instruments** 2-item Patient-provider communication 5-item COVID-19 impact (changes in financial and resource-access) 3-item COVID-19-specifc threat sensitivity (Conway et al., 2020) 3-item Cancer progression risk perception (perceived risk given COVID-19-related treatment changes) 8-item Fear of cancer progression (FCP) adapted from FCRI-SF (Fardell et al., 2017) Generalized anxiety and depression PROMIS Short Form Anxiety 4a and Depression 4a (Cella et al., 2010) 1-item Sleep quality from PSQI (Buysse et al., 1989) 1-item Quality of life from FACT-G (Cella et al., 1993) **Inclusion criteria:** women who (1) were diagnosed with DCIS, LCIS, or invasive breast cancer, (2) whose cancer surgery was postponed as a result of the COVID-19 pandemic, and (3) spoke English.	Sociodemographic characteristics: age race, ethnicity, gender, level of education, income cancer history and treatment	Correlations between FCR and communication satisfaction, perceived risk (concern), perceived risk, generalized anxiety, depression, sleep quality, and quality of life. Significant differences between survivors awaiting surgery and those post-surgery regarding the estimation of risks of cancer progression.	Limitations: Sample size was relatively small Participants were generally financially secure, highly educated, and did not report being severely impacted by the COVID-19 pandemic
5	[51] China 9th-30th Apr 2020	Multi-center cross-sectional survey Online or paper form	**N = 488** breast cancer survivors referred to radiotherapy	**Instruments** Influence of COVID-19 Pandemic on RT 12-item Fear of Progression Questionnaire ⁃ Short Form (FoP-Q-SF) Hospital Anxiety and Depression Scale (HADS) Quality of life (QoL) during pandemic - EORTC QLQ-C30 - five functional scales (physical, role, emotional, cognitive and social function) and global QoL **Inclusion criteria** confirmed pathological diagnosis of BC referred to RT during the COVID-19 pandemic	Age Gender Employment status Level of education Marital status Stage of tumor Type of recurrent or metastatic breast cancer Surgery Chemotherapy Endocrine therapy RT procedure Influence of RT schedule Change	50% of the patients who had to interrupt RT experienced high levels of FCR. - interruption of RT is an independent predictor of FCR, but not for the postponement of RT. strong negative association between all 5 sub-components of quality of life (physical, role, emotional, cognitive, and social) and FCR, and both emotional (r = −.103, *p˂.001*) and social functioning (r = −.052, *p=.006*) were found to be independent predictors for high levels of FCR	Limitations Possible recall bias Limited information regarding demographic variables No control group from the pre-pandemic period
6	**Gultekin et al., 2020** 16 European countries (France, UK, Italy, Spain, Greece, Turkey, Czech Republic, Germany, Netherlands, Denmark, Poland, Serbia, Hungary, Belarus, Ireland, Finland) 1st May – May 31, 2020	Prospective survey	**N = 1251** Gynecological cancer online and hard copy assessment	**Instruments** Covid-19-related sections 1 item assessed the patients' concern about the progression of cancer due to the cancellation or postponement of treatment/follow-up HADS – anxiety and depression Two open-ended questions: 1 “What is the most challenging problem in this period?” 2 “Message that you want told to share about Covid-19 pandemic with ESGO, ENGAGe and Other International Organizations”	No specification for stage, type, and histology of cancer Stage of treatment Type 1 = diagnosis of primary, or recurrent cancer scheduled for surgery Type 2 = receiving chemo, and/or radiotherapy for primary or recurrent disease Type 3 = Under routine oncologic follow-up Patients with previous psychiatric disorders, diagnoses unrelated to cancer and receiving medical treatment (e.g., bipolar, schizophrenia) were excluded	Cancer related 71% of the assessed patients indicated that they were concerned about their cancer progression due to the possible cancellations and/or postponements of their treatment/follow-up FCR was investigated as a risk factor for abnormal HADS anxiety and HADS depression scores	1-item assessing the concern of concern progression due to cancellation/postponement of treatment/follow-up
7	[54] Australia 22nd July – August 19, 2020	Cross-sectional study Online assessment	**N = 394** hematological cancer Most common lymphoma and leukemia	**Inclusion criteria** Adults older than 18 years of age Currently or previously diagnosed with hematological cancer	Demographic variables: age, gender, postcode (residence) marital status, education level, employment status, Medical characteristics: Cancer care experience Financial concerns Concerns about the impact of COVID-19 on their own health and their perceived risk of contracting COVID-19 Psychological distress 10-item Kessler assessment, Unmet supportive care needs Health system and Information needs Patient care and Support needs Supportive Care Needs Survey (SCNS-SF34) Fear of Cancer Recurrence Inventory (FCRI)	35% had elevated scores on the Kessler Psychological Distress scale 9% severe distress 32% -experiencing at least one unmet moderate or high need All respondents – some degree of FCR 95% clinical levels of FCR Psychological distress and concern about the impact of COVID-19 on cancer management were associated with greater FCR during the pandemic, explaining 28% of variance in FCR No significant moderators of this relationship identified	Exclusion of patients with diverse cultural and linguistic background due to limited knowledge of English Potential bias due to the use of the self-selection recruitment method No possibility to establish causation pathways due to study design High heterogeneity of hematological cancer forms Did not examine the possible effect of medical care offered to some patients via telehealth
8	[52] Wuhan, China 15–17 April 2020	Cross-sectional	**N = 326** Breast Digestive system Lung Other	**Instruments** Fear of disease progression and psychological stress Fear of Progression Questionnaire-Short Form (FoP-Q-SF), Self-Rating Anxiety Scale (SAS), Self-Rating Depression Scale (SDS)	Gender Age Marital status, Reproductive history, Educational level, Income, Level of concern about the COVID-19 outbreak, Cancer type, Co-morbidity (Yes-No), Living style (Alone-With Family/friends), Impact of COVID-19 outbreak on cancer treatment (normal-delayed-interrupted)	86.5% of the assessed cancer patients indicated elevated levels of fear of cancer progression, 67.5% elevated levels of anxiety, and 74.5% elevated levels of depression FCR was found to be significantly associated with: educational level income cancer diagnosis deep concern about Covid-19 treatment disturbance Lung **˂.001** Delay or interruption of cancer treatment **˂.001** Deep concern about Covid-19, **˂.001**	- did not use cut-points to indicate the percentage of patients who have experienced low, medium, high levels of FCR
9	[40]	Cross-sectional study carried out face-to-face in multiple centers, paper pencil assessment	**N = 1585** cancer patients from 7 Romanian oncological hospitals ages 17-87	**Instruments** Structured questionnaire based on the model proposed by the WHO Regional Office for Europe 64 questions related to knowledge, attitudes, and practices (KAP) related to Covid-19 - level of distress about contracting the infection, - current knowledge about the disease, - perception of the threat of coronavirus and – the impact of the pandemic upon cancer outcome, - methods of prevention used and their efficacy, - level of trust in the capability of medical staff to manage COVID-19 - level of trust in different sources of information regarding coronavirus	Socio-demographic information	- 32.6% very worried about getting infected or developing Covid-19 and 61.8% feared both cancer evolution and Covid-19 equally - cancer patients with lower income and higher education were more worried about contracting Covid-19 - 26.5% of the patients were more concerned about the cancer progression than fearing a possible infection with Covid-19 - 28.3% indicated that the measures taken during the pandemic negatively impacted the cancer trajectory	- low number of items addressing FCR and FCP -high levels of heterogeneity regarding different types of cancer

## 10 Results

We have identified 9 studies that were published during the Covid-19 pandemic period up till the October 1, 2021. The 9 studies investigated a total number of 4,831cancer survivors during either in the first (2020) or the second (2021) year of the Covid-19 pandemic. Of the 9 included studies: 5 investigated fear of cancer recurrence or progression in breast cancer survivors (N = 810), one investigated a sample of Romanian adult cancer patients (N = 2050), more than a quarter of them being diagnosed with breast cancer (26,30%), and the rest with oncological pathologies such as digestive, lung or others, one investigated a sample of survivors diagnosed with breast, digestive, lung or other types of cancer (N = 326), and the other two studies investigated other oncological pathologies, including gynecological cancer survivors (N = 1251) and hematological cancer survivors (N = 394).

First, we will briefly present the major results obtained by the studies focusing on breast cancer survivors, followed by the studies including patients diagnosed with gynecological, hematological, breast/digestive/lung/other forms of cancer.

## 11 Breast cancer

1 **Kim and Kim's** [48] investigation included 154 breast cancer patients, using both quantitative and qualitative methods. Fear of cancer recurrence was measured with the Korean version of the Fear of Cancer Recurrence Inventory (K-FCRI). These scales assessed FCR across seven sub-domains: triggers, severity of FCR, psychological distress associated with FCR, coping strategies associated with FCR, functional impairments due to FCR, insight, and reassurance. The global scores that could be obtained on his measure ranged between 0 and 168, higher scores indicating higher levels of FCR. FCR global scores were used to offer a complete picture of the fear of cancer recurrence, while the FCR severity sub-score was used to investigate participants' clinical status. Regarding the severity sub-factor of FCR, scores ranging between 0 and 15 indicated minimal levels of FCR, between 16 and 23 problematic levels, and scores over 22 indicated clinically significant levels of FCR due to the changes in the treatment plan during the Covid-19 pandemic [48].

According to the results, the mean of the global score of FCR on this sample was 84.31 (SD ± 24.23). The average severity of FCR for the entire sample was 19.12 (SD = 6.25). Furthermore, 35.7% of the participants assessed in this study attained severity scores higher than 22, suggesting that over one third of the participants experienced a clinically significant level of fear of cancer recurrence. This result was compared with the results obtained on similar populations, assessed with the same instrument in non-Covid-19 periods, and the results indicated that the number of the participants assessed in the Kim and Kim's [48] study, who reported clinically significant values of FCR (meaning 22 points) was twice as high. However, as the authors have emphasized, these differences have to be interpreted with caution because the previous study to which they compared their results involved older participants, the period of time between diagnosis and assessment was longer, and was conducted before the pandemic. According to this study, FCR was significantly higher among participants that were not married (F = 3.649, *p = .028*), childless (t = −2.043, *p = .043*) less financially potent participants (F = 4.259, *p = .016*) [48].

A very important finding of this study is that unmarried, childless and less financially potent breast cancer survivors may be more prone to experience significantly higher levels of FCR due to the implications related to the Covid-19 pandemic [48].

2 **Koral and Cirak** [33] investigation was conducted on 82 Turkish, non-metastatic breast cancer survivors indicated that 84.1% of the participants experienced high levels of FCR, and that there existed a statistically significant negative correlation between fear of cancer recurrence and resilience (r = −0.316, *p=.004*), as well as spiritual well-being (r = −0.329, *p=.003*). Hierarchical linear regression analysis indicated that SWB significantly affects FCRI-SF Scores (SC Beta = 0.255, p = .041), while the mediation analysis revealed that SWB plays a partial mediating role in the relationship between BRS and FCR. Variance analyses did not indicate any statistically significant differences in FCR depending on the assessed demographic variables [33].

A very important limitation of this study was that it excluded cancer patients older than 50 years of age.

3 **Massicotte et al.'s** [49] study was conducted on 36 non-metastatic breast cancer survivors. According to the study, 52.8% of the participants indicated clinical levels of FCR, anxiety (44.4%), and insomnia (41.7%). Almost two thirds of the participants (63.9%) reported at least one stressor associated with the implications of the COVID-19 pandemic, the most intense concerns being caused by: difficulty obtaining food, medicine, and essentials (8.3%), postponement or cancellation of cancer treatment (19.4%), possible changes in the oncological care (11.1%), postponement of medical investigations (11.1%). Interestingly, the results of this study indicated that the number of stressors significantly correlated with anxiety [τ(32) = 0.34, *p = .01*], depression [τ(32) = 0.33, *p = .02*] and insomnia, [τ(32) = 0.33, *p = .01*], but not with Fear of Cancer Recurrence [τ(32) = 0.12, *p = .38*] (this result has to be interpreted with utmost precaution, due to the extremely low number of participants). However, higher levels of concerns regarding the implication of Covid-19 pandemic was significantly associated with FCR [r(32) = 0.59, *p < .001*], anxiety [r(32) = 0.54, *p = .001*], depression [r(32) = 0.36, *p = .04*], and insomnia [r(32) = 0.55, *p=.001*]. These results indicate that not necessarily the number of the Covid-19 related stressors, but the level of concerns is important especially from the point of view of fear of cancer recurrence [49].

The study has several limitations, one of which being the extremely low number of participants and that it was conducted at the very beginning of the pandemic.

4 **Soriano et al.'s** [50] study was conducted on 50 non-metastatic, female breast cancer survivors whose breast surgery was postponed due to the Covid-19 pandemic. 26% of the participants reported elevated fear of cancer progression (FCP) of clinical intensity. If results be interpreted according to Simard and Savard's (2009, 2015) recommendation, 60% of the assessed patients fell into the clinically significant intensity range of FCR. 15% of participants reported low levels of quality of life, and 4% high levels of anxiety, and 2% severe forms of depression.

Correlation analysis indicated that communication between patient and health-care providers regarding possible delays in surgery due to pandemic-related was significantly associated with lower levels of COVID-19 impact (r = −0.50, p < .01), lower perceived risk of cancer progression (r = −0.32, p < .05), lower FCP (r = −0.36, p < .05), and fewer depression symptoms (r = −0.29, p < .05). Furthermore, higher impact of COVID-19 scores significantly correlated with greater perceived risk of cancer progression (r = 0.29, p < .05), more generalized anxiety symptoms (r = 0.35, p < .05), and lower quality of life (r = −0.39, p < .01).

The study also found a very strong inter-correlational pattern between perceived risk (concern), the perceived risk estimate (0–100%), and FCP (rs.64–0.69, p < .01) [50].

FCP was significantly associated with the following psychosocial variables: communication satisfaction (r = −.359, *p˂.05*), perceived risk (concern) (r = 0.689, *p˂.001*), perceived risk (0–100%) (r = 0.659, *p˂.001*), generalized anxiety (r = 0.681, *p˂.001*), depression (r = 0.614, *p˂.001*), sleep quality (r = 0.374, *p˂.001*), and quality of life (r = 0.374, *p˂.01*).

Differences due to surgery status indicated that survivors awaiting surgery also estimated that they had higher risks of cancer progression (M = 40%) than those post-surgery (M = 25%) [0–100%, t(46) = 1.73, p = .091], with moderate effect sizes (g = 0.53) [50].

The major limitations of this study were represented by the relatively small sample size, and by the fact that most participants were generally financially secure, highly educated, and did not report being severely impacted by the COVID-19 pandemic.

5 **Xie et al.'s** [51] study investigated 488 breast cancer survivors referred to radiotherapy, and who had to postpone or interrupt treatment due to the Covid-19 pandemic. The authors hypothesized that fear of cancer recurrences in these patients would be negatively impacted due postponement of RT treatment. Thus, this study investigated the prevalence of FCR, and some of its predictors in breast cancer patients referred to RT, but who had to postpone the beginning of treatment due to the pandemic.

According to the results of this study [51], 17.2% of the entire sample reported high levels of FCR, the two most common sources of fear in the sample being: worrying if medications could damage the body, and worrying about the future of family members. Moreover, 50% of the patients who had to interrupt RT experienced high levels of FCR. Hierarchical multiple regression models further indicate that interruption of RT is an independent predictor of FCR, but not for the postponement of RT. According to the authors the level of awareness regarding the implications of the treatment-delay is a very important predictor of FCR. Their results indicate that the levels for FCR in those patients who believed to have delayed RT plans, but actually did not, was much higher (31.5%) than in the normal (13.2%) or real delay group (11.4%). In line with previous studies, Xie et al.'s [51] investigation indicates a strong negative association between all 5 sub-components of quality of life (physical, role, emotional, cognitive, and social) an FCR, and both emotional (r = −0.103, *p˂.001*) and social functioning (r = −0.052, *p=.006*) were found to be independent predictors for high levels of FCR [51].

Limitations of this study include: possible recall bias, limited information regarding more detailed demographic variables, no control group from the pre-pandemic period.

6 **Gheorghe et al.** [40] study was conducted on 1585 Romanian cancer patients under treatment at the time of the assessment, with ages ranging between 17 and 87 (M = 60, SD = 11.4). The sample included patients diagnosed with breast (26,30%), digestive, lung or other types of cancer, from seven Romanian medical centers who were assessed regarding their: levels of distress about contracting the Covid-19 infection, knowledge about the Covid-19 and its implications (perception of the threat of coronavirus), the possible impact of the pandemic upon cancer outcome, methods of Covid-19 prevention and their efficacy, trust in the capability of medical staff to manage Covid-19 and trust in different sources of information regarding Covid-19. The results of this study indicate that 32.6% of the assessed patients reported high levels of worry about getting infected or developing Covid-19 and 61.8% feared both cancer evolution and Covid-19 equally, 26.5% of the patients were more concerned about the cancer progression than fearing a possible infection with Covid-19, and 28.3% indicated that the measures taken during the pandemic negatively impacted the cancer trajectory. Moreover, cancer patients with lower income and higher levels of education were more worried about contracting Covid-19 [40].
7 **Chen, Wu, Jiang, Zhang, Peng, Hu, et al.'s** [52] study investigated 326 patients diagnosed with breast/digestive/lung/other types of cancer. According to this study, 86.5% of the assessed cancer patients indicated elevated levels of fear of cancer progression, 67.5% elevated levels of anxiety, and 74.5% elevated levels of depression. However, since it did not use cut-point-interpretations, the paper does not mention the percentage of patients how reported low, moderate and high levels of FCR. According to this study, FCR was significantly associated with educational level (*p=.001*), income (*p=.001*), cancer diagnosis (*p=.001*), deep concern about Covid-19 (*p=.001*), and treatment disturbance (*p=.001*). Regression analyses showed that the factors that were positively associated with FCR during COVID were: treatment delay [B(95%CI) = 1.45, *p=.001*], treatment interruption [B(95%CI) = 1.98, *p=.001*], deep concern about COVID-19 [B(95%CI) = 2.55, *p=.001*], and lung cancer diagnosis [B(95%CI) = 0.087, *p=.020*]. Self-rated anxiety (SAS) scores were positively associated with treatment delay [B(95%CI) = 10.49, p = .001], treatment interruption [B(95%CI) = 11.38, *p=.001*], deep concern about COVID-19 [B(95%CI) = 1.86, *p=.001*], and type of cancer (lung diagnosis) [B(95%CI) = 0.92, p = .009], and it negatively associated with levels of education [B(95%CI) = -0.95, *p=.001*]. Self-rated Depression (SDS) scores were positively associated with treatment delay [B(95%CI) = 9.74, *p=.001*], treatment interruption [B(95%CI) = 10.92, *p=001*], deep concern about COVID-19 [B(95%CI) = 0.92, *p=.028*], and marital status (unmarried) [B(95%CI) = 2.62, *p=.025*], and it negatively with higher income [B(95%CI) = -0.94, *p=.023*] [52]. One of the major limitations of the present study is that it does not mention the time elapsed since diagnosis, and did not use cut-points to indicate the percentage of patients who have experienced low, medium, high levels of FCR.
8 **Gultekin et al., (2020)** investigation involving 1251 gynecological cancer patients from 16 European countries indicated that 71% of the assessed patients reported being concerned regarding their fears of cancer progression due to the possible cancellations and/or postponements of their treatment/follow-up. Moreover, even if the majority (73.2%) of the assessed gynecological cancer survivors considered that oncological patients in general were exposed at higher risk to be infected with Covid-19, only 17.5% of them reported of being more afraid of a possible Covid-19 infection than being afraid of their oncological condition, and 53.1% were afraid that they might contract Covid-19 from health care sites, while receiving treatment for their oncological ailments. Furthermore, according to the results of this study, the only risk factor found for higher levels of fear of Covid-19 than of fear related to the oncological diagnosis was age older than 70 years.

This study [53] also indicated that on the HADS anxiety and depression instrument, 35.3% of the assessed patients had abnormal levels of anxiety and 30.6% abnormal levels of depression scores. However, multivariate logistic regression analysis did not indicate that being afraid of cancer progression would have any significant effect on patients' anxiety or depression levels.

The two open-ended questions also offered very interesting results regarding the fears and worries of gynecological cancer patients during the Covid-19 pandemic. The first question “What is the most challenging problem in this period?” was answered by 623 of the assessed participants in the study, 44% of which reported that the uncertainty produced by the Covid-19 and its implications represented a significant concern for them, while only 2% of the assessed patients reported worry due to possible financial concerns induced by the pandemic. Regarding the second open-ended question “Message that you want told to share about COVID-19 pandemic with ESGO, ENGAGe and Other International Organizations”, 65% of the respondents considered that it should be emphasized that cancer is more lethal than Covid-19, and that something should be done to better protect cancer patients. Summarizing the findings of this study [53]:

(1) Most cancer patients considered that having cancer was a major risk factor for contracting Covid-19, less than 1/5 of the assessed patients were more afraid of Covid-19 than cancer, and most of these patients were older than 70 years of age.
(2) Even if most of the assessed patients were afraid of the possibility of being infected with Covid-19, their major concern remained the potential implications regarding the progression of their oncological disease due to the disruption of treatment, follow-ups etc. during the pandemic.

However, this study also has some serious limitations, as: the assessment of FCR with one single item, which did not permit the establishment of low, medium and clinically significant levels of FCR.

9 **Zomeredijk et al.'s** [54] study was conducted on 394 hematological cancer - the most common forms being lymphoma (34%) and leukemia (27%). The major results of this investigation indicate that 35% of the sample reported elevated scores on the Kessler Psychological Distress Scale, 9% of them attaining severe levels of distress. 32% of the participants experienced at least one unmet moderate or high need. The most frequently cited unmet needs were: (1) being able to access professional counseling (15%), (2) obtaining information about test results as soon as possible (15%), and (3) being treated like a human being (15%).

All of the patients who had completed treatment and were in remission (n = 134), reported some degree of FCR, 95% of them reporting clinical levels of FCR. Psychological distress and concern about the impact of COVID-19 on cancer management were found to be significantly associated with greater FCR during the pandemic among hematological cancer patients in remission, explaining 28% of variance in FCR [54].

The major limitations of the present study were: potential bias due to the self-selection recruitment method, high heterogeneity of hematological cancer forms, did not examine the possible effect of medical care offered to some patients via telehealth.

Thus, this scoping review highlighted socio-demographic and psychological variables significantly associated with FCR during the COVID-19 pandemic. FCR was significantly higher among patients who were unmarried or without children, being negatively associated with monthly income [48] and educational level [52], participants who generally were financially secure and highly educated did not report being severely impacted by the COVID-19 pandemic [50]. However, Romanian cancer patients with lower income and higher education reported higher levels of concern about contracting Covid-19, suggesting that income and education may play an important role in the relationship dynamic between FCR and COVID-19 [40]. Moreover, age was the only risk factor for higher levels of fear of Covid-19 than of fear related to the oncological diagnosis, patients older than 70 years being more concerned about COVID-19 than about their cancer progression (Gultekin et al., 2020). Some studies [33,54] did not identify statistically significant differences in FCR depending on the assessed demographic variables, while others [49,51] presented limited information regarding results about the investigated demographic variables.

Regarding psychological variables significantly associated with FCR during the COVID-19 pandemic, higher levels of concerns regarding the implication of the pandemic were significantly associated with FCR, anxiety, depression, and insomnia [49]. Other studies found correlations between FCR and satisfaction with the quality of communication with the medical staff, perceived risk, impaired sleep quality and quality of life [50], and elevated levels of anxiety and depression [52]; Gultekin et al., 2020; [51]. Therefore, almost all studies included identified elevated levels of FCR during the COVID-19 pandemic [54], with patients also reporting fear, unmet needs and psychological distress about the impact of COVID-19 in the management of the oncological treatment. Moreover, beside concerns about negatively-impacted cancer treatment trajectory, patients fear both the evolution of the cancer and a possible infection with Covid-19 [40], psychological distress about the impact of COVID-19 on cancer management being associated with greater FCR during the pandemic, explaining 28% of variance in FCR [54].

## 12 Conclusions

As we have seen in the introductory part of this paper, the diagnosis with cancer, and the considerably changed life-conditions seriously impact most oncological patient's life. FCR can be problematic well into the years after cancer has been successfully treated [55], and frequently accompanies the unfolding of the (mal)adaptive oncological survivorship trajectory [18]. Even if mostly rational, when FCR exceeds the healthy threshold, it may further increase the psychological sensitivity of oncological patients, impacting their physical and psychological state.

In normal life conditions the stable risk factors for increased FCR for most forms of cancer are: younger age, presence and severity of symptoms, side-effects of treatment, pain, treatment with chemotherapy, previous traumatic encounters, psychological disorders such as: anxiety, mood disorders, and high levels of neuroticism. Moreover, disruptions in the access to health-care services, pending medical appointments, new or changing side-effects of treatment, intensification of pain may further exacerbate FCR.

Literature indicated that the Covid-19 pandemic severely affects cancer survivors both directly (the high susceptibility to contract Covid-19), and/or indirectly (disruption of treatment, impeded access to health care, delays in diagnosis, etc.) [56].

Thus, the major aim of the present paper was to review the currently available literature which examines factors that are associated with higher levels of FCR in breast cancer and other oncological pathologies survivors during the Covid-19 pandemic.

The low number of studies investigating the specific issue of FCR during the Covid-19 pandemic, including a large variety of survivors from the point of view of cancer type, age-range (several studies included only patients younger than 50 years of age), geographic area (Asia, Australia, Canada, Europe, USA), variety of methodological approaches (quantitative, mixed), variety of scales used (standardized and validated vs. non-validated, reduced item-number scales, single questions), etc. does not allow for the extraction of a large number of firm and cross-situationally stable conclusions. However, as expected, one of the major conclusions of this study is, that during the Covid-19 pandemic, most of the assessed patients experienced significantly higher levels of distress than similar groups of cancer patients assessed in non-Covid-19 times. Moreover, the percentage of those who experienced significantly higher levels of FCR or FCP were also higher in all studies investigating this aspect, higher levels of FCR being correlated with psychological distress, and concerns regarding the impact of Covid-19: disturbances in treatment (delays and interruption), dysfunctional communication with the medical stuff, concern about the implications of the Covid-19 regarding access to food, medications, being the most frequently encountered worries. Another essential aspect that emerged in one of the studies [49] refers to the fact that maybe not the number of the Covid-19 related stressors, but the level of concerns weighs more in the perceived risk of cancer progression. This result may further become the objective of future investigations.

Among the most common correlates of FCR during the Covid-19 pandemic were: marital status (in one study, unmarried patients experienced significantly higher levels of FCR), childlessness, low financial status [48], education level (patients with lower levels of education experienced significantly higher levels of FCR), lower income, cancer diagnosis (patients diagnosed with lung cancer experienced higher levels of FCR) [52], resilience and spiritual well-being proved to be protective factors in the development of FCR [33], generalized anxiety, depression, sleep quality, quality of life [50], quality of emotional and social life [51], psychological distress and concern regarding the impact of Covid-19 on cancer management [54].

A Bandinelli et al.'s (2021) study states, in the case of cancer patients during the Covid-19 pandemic we can speak not of clearly-delimited, well-defined types of anxieties and fears. But of a sum of fears, that overlap, and potentially potentate each other [57].

Summarizing, studies show that FCR is associated with dysfunctional reactions, impaired well-being, quality of life and professional functioning, anxiety, depression and posttraumatic stress disorders [22,23]. The COVID-19 pandemic can exacerbate the already existing FCR in cancer patients, given the access restrictions to follow-up and treatment, the isolation restrictions imposed and the possibility that the medical system becomes overworked. Therefore, the treatment's trajectory, investigations, and the psychological health of people suffering from breast cancer are negatively affected during these times. Based on the studies presented in this scoping review, during this pandemic period, FCR is correlated with cancer patients' concerns about possible delays or interruptions in treatment plans and about dysfunctional communication with medical staff [50]. Studies identified elevated levels of FCR during the COVID-19 pandemic, as well as elevated levels of anxiety and depression (Gultekin et al., 2020; [49,51]. The COVID-19 pandemic is a context that may worsen the already existing FCR, cancer patients reporting now additional psychological distress about a possible infection with the virus besides the fear related to cancer evolution during this period [40,54].

Scoping reviews often lay ground for more complex analyses on a topic (e.g., systematic reviews, meta-analyses) in situations when such investigations do not yet exist. They offer the possibility to systematically map the literature investigating a specific issue [44], and to summarize evidence, and identify possible knowledge gaps [45]. Nevertheless, scoping reviews have their limitations that have to be taken into account. First of all, they usually review a broad research topic, which may lead to similarly broad findings [58]. Another limitation is that they do not qualitatively assess the included studies [45]. The authors considered that due to the salience of the topic and the scarcity of published studies, the scoping review was an appropriate method to investigate the major objectives of the present study. Thus, due to the limitations of this method we recommend that the results of this study to be taken as evidencing the necessity to further investigate FCR during the Covid-19 pandemic, and be considered a starting point for future, more complex and rigorous investigations that could further inform policy.

Previous findings in breast cancer patients have linked attentional bias to distress, attentional bias predicting more severe depressive and anxiety symptoms post-diagnosis [59,60]. Hospital study results revealed that both high-fearful and low-fearful breast cancer survivors showed more interference by cancer words than the healthy controls indicating that the specific type of metacognitive thinking underlying fear of cancer recurrence may be more akin to anxiety disorder [25]. In addition, an intervention program targeting cognitive bias modification for fear of cancer recurrence in breast cancer survivors, adapting a program initially developed for anxiety disorders, has shown limited preliminary efficacy [61]. Other studies reported inconsistent data with no clear evidence of attention biases associated with fear of cancer recurrence, suggesting that care should be taken in applying such treatment components in clinical cancer care [62]. We propose that future studies investigating FCR invest significant attention in the study of these factors.

Since the future evolution of the pandemic is highly uncertain worldwide, the aggravation of the mental health state of breast cancer and other oncological pathologies survivors during the pandemic should become an issue of crucial importance for all levels of health care services. The immediacy with which the concerted efforts of medical stuff and mental-health care practitioners address FCR and FCP during the Covid-19 period (e.g., facilitation of access to medical care, the maintenance of normal treatment schedules, increased access to mental health care practitioners, financial help for persons with low financial status, etc.) would not only alleviate clinical and/or psychological/psychiatric symptoms but also increase cancer survivorship.
